# Characterizing Gray matter atrophy patterns associated with accelerometer-measured sedentary behavior: a population-based study

**DOI:** 10.1007/s11682-025-01054-1

**Published:** 2025-09-30

**Authors:** Minle Tian, Xiaolei Han, Ming Mao, Xiaomeng Li, Yi Dong, Jiahao Ding, Qinghua Zhang, Shi Tang, Xiaojuan Han, Lin Song, Tingting Hou, Lin Cong, Yifeng Du, Chengxuan Qiu, Yongxiang Wang

**Affiliations:** 1 Key Laboratory of Endocrine Glucose & Lipids Metabolism and Brain Aging, Department of Neurology, Ministry of Education, Shandong Povincial Hospital, Shandong First Medical University, No. 324 Jingwuweiqi Road, Jinan, Shandong 250021 P.R. China; 2 Shandong Institute of Brain Science and Brain-inspired Research, Medical Science and Technology Innovation Center, Shandong First Medical University & Shandong Academy of Medical Sciences, Jinan, Shandong P.R. China; 3 Aging Research Center, Department of Neurobiology, Care Sciences and Society, Karolinska Institutet-Stockholm University, Stockholm, Sweden

**Keywords:** Accelerometer, Sedentary behavior, Voxel-based morphometry, Gray matter, Population-based study

## Abstract

Evidence has linked self-reported sedentary behaviors with dementia and cognitive impairment; however, the underlying mechanisms remain poorly understood. We investigated the associations of accelerometer-measured sedentary behavior patterns with gray matter atrophy patterns in rural-dwelling older adults, while taking into account the manner in which sedentary time is accrued (in short or long bouts). This community-based study involved 911 dementia-free older adults (age ≥ 60 years, 59% women) who participated in both ActiGraph and brain MRI substudies within MIND-China (2018–2020). Sedentary behavior parameters (total sedentary time, mean sedentary bout duration, and sedentary breaks) were recorded with accelerometers. Regional gray matter volumes (GMV) were measured using voxel-based morphometry (VBM) methods. Data were analyzed using the general linear regression models, restricted cubic spline curves, and VBM analysis. There was an inverted U-shaped association between daily sedentary time and GMV in temporal, cingulate, and medial temporal cortex, while longer mean sedentary bout duration was linearly related to decreased GMV in total, frontal, temporal, insula, cingulate, and medial temporal cortex. Greater daily time spent in light or moderate-to-vigorous physical activity (LPA and MVPA) was correlated with larger insula GMV. The VBM analysis suggested that prolonged daily total sedentary time and mean sedentary bout duration were significantly associated with smaller GMV in extensive brain regions, especially in thalamus and insula. In conclusion, gray matter atrophy associated with sedentary behavior in older adults is characterized by reduced GMV in global, frontal, temporal, medial temporal, and cingulate cortex, especially in the insula and thalamus regions.

**Supplementary Information:**

The online version contains supplementary material available at 10.1007/s11682-025-01054-1.

## Introduction

Dementia affects over 50 million people worldwide and is one of the leading causes of disability-adjusted life years, a measure that combines years of life lost due to premature death and years lived with disability (Livingston et al., 2017). The meta-analysis and the 2024 report of the Lancet Standing Commission both identified physical inactivity as a major modifiable risk factor for dementia, with a weighted population attributable fraction (PAF) being 2% and 7.3%, respectively (Stephan et al., 2024; Livingston et al., 2024). Indeed, regular physical activities in both light and moderate-to-vigorous-intensity have been frequently linked with structural and functional brain health (Gu et al., 2020; Raji et al., 2024; Spartano et al., 2019). By contrast, although previous studies have reported the associations of sedentary behavior with cognitive impairment and dementia(Wang et al., 2019; Yan et al., 2020), evidence linking objectively-measured sedentary behavior patterns with brain health remains relatively limited (Collins et al., 2023; Wheeler et al., 2017).

Sedentary behavior is defined as any waking activity performed in a sitting or reclining posture with an energy expenditure of ≤ 1.5 metabolic equivalents (METs)(Tremblay et al., 2017). Several systematic reviews and meta-analyses, primarily based on observational studies, have reported that lower levels of sedentary behavior are associated with better performance in global cognitive function (Falck et al., 2017; Rojer et al., 2021). A recent meta-analysis found that increased time spent in sedentary behavior was associated with a higher incidence of dementia (Yan et al., 2020). In addition to total sedentary time, patterns of sedentary behavior-such as the average duration of prolonged sedentary bouts and the frequency of interruptions (sedentary breaks)-seem to be associated with cognitive outcomes. A prospective cohort study from the UK Biobank observed that self-reported prolonged sedentary time was associated with an increased risk of dementia (Huang et al., 2022). Therefore, the accumulation of sedentary time in either a few long bouts or many short bouts represents two distinct patterns of sedentary behavior that may have different associations with adverse health outcomes (Bellettiere et al., 2019; Diaz et al., 2017a; Dunstan et al., 2021). We previously reported that prolonged uninterrupted sedentary time was associated with poor global cognition, memory, and verbal fluency among rural older adults, partially mediated via structural brain magnetic resonance imaging (MRI) markers such as white matter hyperintensities (WMH), white matter, ventricular, and hippocampal volumes (Han et al., 2023). However, the patterns of gray matter atrophy in predefined brain regions associated with accumulating sedentary time in prolonged and uninterrupted bouts in the general population settings are poorly defined.

Voxel-based morphometry (VBM) is a powerful tool in characterizing subtle structural changes in brain regions that are linked to a range of brain disorders. The Irish Longitudinal Study on Ageing found that high-sedentary individuals (> 8 vs. ≤8 h/day) showed lower hippocampal volumes and increased WMH (Maasakkers et al., 2021). Another study found that the medial temporal lobe (MTL) thickness was inversely correlated with self-reported time spent in daily sitting (Siddarth et al., 2018). However, most of the previous studies have focused on the relationship between daily sedentary time and global and certain regional brain atrophy, whereas the associations of gray matter atrophy patterns with patterns of sedentary behavior accumulation have rarely been investigated. Characterizing global and regional grey matter atrophy associated with sedentary behavior patterns may help develop interventions to improve brain health in aging, thus, reducing the risk or delaying the onset of dementia disorders.

In the current study, by using data from the Multimodal Interventions to delay Dementia and disability in rural China (MIND-China) ActiGraph and MRI substudies, we sought to characterize gray matter atrophy patterns associated with sedentary behavior in a rural older population. Specifically, we aimed to (1) examine the associations of sedentary behavior parameters with the gray matter volume (GMV) in various brain regions (e.g., frontal, cingulate, and medial temporal cortex) and (2) identify the gray matter atrophy patterns associated with sedentary behavior patterns.

## Methods

### Study design and participants

**Fig. 1:**
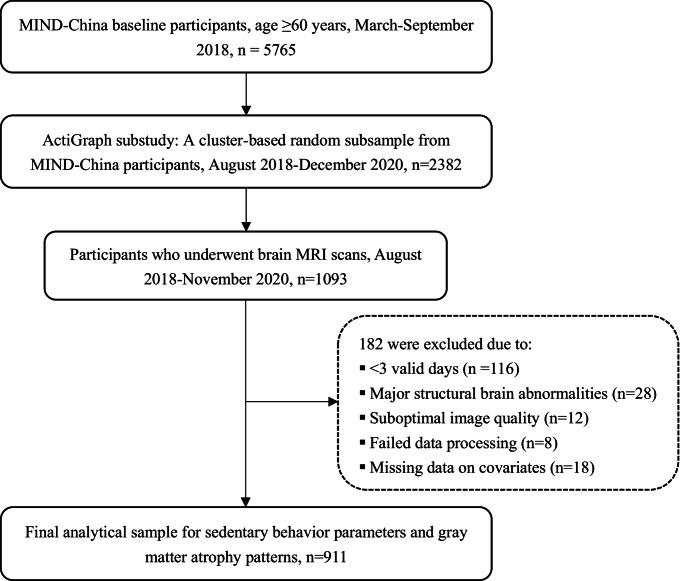
Flowchart of study participants. MIND-China, multimodal interventions to delay dementia and disability in rural China; MRI, magnetic resonance imaging

This population-based cross-sectional study utilized baseline data from the MIND-China ActiGraph and MRI substudies, as previously reported (Han et al., 2023; Wang et al., 2022). In brief, in March-September 2018, a total of 5,765 participants who were aged 60 years or older and Living in 52 villages in Yanggu County, Shandong, undertook the multidisciplinary assessment for MIND-China. Of them, using a cluster (village)-based randomized approach, a subsample comprising 2382 individuals who were free of dementia and major mental health problems were invited to participate in the ActiGraph substudy in August 2018-December 2020 (Han et al., 2022). Of these, 1093 individuals agreed and accomplished the structural brain MRI scans in Southwestern Lu Hospital. Of these, 182 persons were excluded due to < 3 valid days of wearing the accelerometer (*n* = 116), major structural brain abnormalities (*n* = 28), suboptimal image quality (*n* = 12), failed data processing (*n* = 8), and missing data on covariates (*n* = 18), leaving 911 participants for the current analysis. Figure 1 shows the flowchart of study participants.

The protocols of the MIND-China and MRI substudy were reviewed and approved by the Ethics Committee at Shandong Provincial Hospital in Jinan, Shandong. Written informed consent was obtained from all participants, or from a proxy (typically a family member) if the participants themselves were unable to provide consent. The MIND-China study was registered in the Chinese Clinical Trial Registry (registration no.: ChiCTR1800017758).

## Data collection and assessments

Trained medical staff collected comprehensive data through face-to-face interviews, clinical examinations, and laboratory tests following a structured questionnaire, as previously reported (Wang et al., 2022). Alcohol consumption and smoking status were categorized as never, former, or current drinking alcohol or smoking. Body mass index (BMI) and arterial blood pressure were measured as previously described (Wang et al., 2022). Hypertension, diabetes, and dyslipidemia were defined as previously described (Han et al., 2021). The detailed description for the assessments and definitions of study variables can be found in the supplementary materials.

## ActiGraph data collection and processing

Participants were instructed to wear a triaxial accelerometer, the ActiGraph wGT3X-BT (ActiGraph, LLC, Pensacola, FL), affixed to an elastic belt on their hip throughout all waking hours for seven consecutive days, removing it only for swimming or bathing (Han et al., 2022). The wear season, accelerometer parameters, and non-wear time were defined as previously described (Han et al., 2022). We used data from participants who wore the device for ≥ 10 h/day for ≥ 3 days (Tudor-Locke et al., 2012). Sedentary behavior, light-intensity physical activity (LPA), and moderate-to-vigorous-intensity physical activity (MVPA) were defined as < 100, 100-1,040, and ≥ 1,041 counts per minute, respectively (Copeland & Esliger, 2009; Han et al., 2023).

Sedentary behavior parameters included total sedentary time, the mean sedentary bout Duration, prolonged sedentary time, the number of breaks per sedentary hour, the usual sedentary bout Duration, and alpha. The number of breaks per sedentary hour was calculated as the total number of sedentary breaks divided by sedentary time in hours per day. Alpha is a unitless measure ranging from 1.3 to 2.1 that characterizes the frequency distribution of sedentary bout durations (Chastin & Granat, 2010). A higher value indicates a pattern of sedentary behavior accumulation with more frequent short bouts (i.e., more frequently interrupted) and fewer long bouts. All these sedentary metrics were described and used in our previous studies (Han et al., 2023; Han et al., 2022; Wang et al., 2022). The detailed assessments and definitions of the sedentary behavior parameters are provided in the Supplementary Materials Methods.

## Structural MRI acquisition and data processing

All eligible participants were scanned on a Philips Ingenia 3.0T MR System (Philips Healthcare, Best, The Netherlands) at the Southwestern Lu Hospital. Sagittal T1-weighted structural images were acquired using the parameters as previously reported (Song et al.,^2023^). For image processing, 3D T1-weighted images were utilized to quantify brain volumes. VBM analysis was conducted using the Computational Anatomy Toolbox 12 (CAT 12; http://dbm.neuro.uni-jena.de/cat.html), integrated within the Statistical Parametric Mapping (SPM) 12 package. This analysis was performed on the MATLAB R2013b platform (https://www.fil.ion.ucl.ac.uk/spm/)(Li et al., 2021). A detailed description of the preprocessing steps for the MRI images can be found in the supplementary materials. These smoothed GMV images were then utilized in the subsequent statistical analyses. The total intracranial volume (ICV) was calculated as the sum of the gray matter, white matter, and cerebrospinal fluid volumes (Song et al., 2023). The GMV of the frontal lobe, temporal lobe, parietal lobe, occipital lobe, insula, cingulate cortex, and medial temporal cortex from modulated images were calculated using the Neuromorphometrics atlas available in SPM12 (Neuromorphometrics, Inc.; https://neuromorphometrics.com)(Hirabayashi et al., 2022). Each gray matter voxel that was not initially assigned to any label was subsequently labeled according to the nearest gray matter label, with a distance Limit of 5mm. In the current study, the total GMV, along with the GMVs of the frontal, temporal, parietal, occipital, insula, cingulate, and medial temporal cortex, were calculated as indicators of regional gray matter atrophy.

### Statistical analysis

Participants were categorized into low and high total sedentary time groups based on a median split of total sedentary time within the study population. We presented the mean (standard deviation, SD) for continuous variables and frequencies (%) for categorical variables. We compared sedentary group differences by using χ^2^ test for categorical variables and two-sample t-test for continuous variables. The association patterns of sedentary parameters, LPA, and MVPA with the GMV of the predefined brain regions were examined using restricted cubic spline (RCS) functions (Wang et al., 2023). We reported the results from models using 3 knots (10th, 50th, and 90th per- centiles) for total sedentary time, mean sedentary bout duration, LPA, and MVPA. The boundary knots were set at the minimum and maximum values of the independent variables, ensuring that the splines would adequately capture the relationships within the observed range of the data. The spline models were used to describe the nonlinear associations. When the association was linear, the general linear model (GLM) was used. In case of non-linear associations, inflection points were identified and defined as the daily hours of sedentary time, LPA, and MVPA at which their relationships with GMV in brain regions changed. Then, we further analyzed the associations of sedentary time, LPA, and MVPA with GMV in brain regions stratified by the respective inflection points.

We reported the results from three models: Model 1 was adjusted for age, sex, education, ActiGraph wear season, accelerometer wear time, and total ICV. Model 2 included all covariates from Model 1, with additional adjustment for BMI, smoking habits, alcohol intake, hypertension, diabetes, dyslipidemia, stroke, and coronary heart disease. Model 3 included all covariates from Model 2, with the addition of MVPA.

For the VBM analysis, the association of GMV in the whole brain with sedentary behavior parameters, LPA, and MVPA was analyzed using multiple linear regression models in the SPM statistical software package, while controlling for age, sex, education, ActiGraph wear time and wear season, total ICV, smoking habits, alcohol intake, and BMI. We performed the aforementioned analyses in the population that showed a linear relationship. Multiple comparisons were corrected using a family-wise error (FWE) corrected *P*-value threshold of 0.05 and a cluster size of at least 200 voxels.

Stata Statistical Software: Release 17 (StataCorp LLC., College Station, TX, USA) or R (R Foundation for Statistical Computing; Vienna, Austria) was used for all analyses.

## Results

### Characteristics of study participants

Of the 911 participants, the mean age was 69.65 years (SD = 4.27), 58.84% were women, and 35.24% had no formal schooling. On average, participants spent 57% of their daily waking time in sedentary behavior, 35% in LPA, and only 8% in MVPA. Participants were divided into low and high total sedentary time groups using a median split of total sedentary time within the study population (≤ 8.06 h as low; >8.06 h as high). Compared to participants with lower total sedentary time, those with higher total sedentary time tended to be older, more likely to be male, and have a higher BMI, hypertension, and history of stroke (*p* < 0.05, Table 1). Additionally, individuals with higher total sedentary time were more likely to have schooling education, smoke, and consume alcohol (*p* < 0.05). Participants in the high-sedentary group wore the device significantly longer than those in the low-sedentary group. Seasonal differences were also observed: a higher proportion of the high-sedentary group wore the device in summer, while more participants in the low-sedentary group did so in autumn. No significant group differences were found in diabetes, dyslipidemia, or coronary heart disease. (Table 1).

**Table 1:** Characteristics of study participants

Characteristics	Total sample (*n* = 911)	Total Sedentary Time		
		Low (*n* = 455)	High (*n* = 456)	*P* Value
Age (y)	69.65 (4.27)	68.92 (4.23)	70.38 (4.19)	< 0.001
Female sex, n (%)	536 (58.84)	325 (71.43)	211 (46.27)	< 0.001
Education, n (%)				< 0.001
No schooling	321 (35.24)	196 (43.08)	125 (27.41)	
Primary school	397 (43.58)	181 (39.78)	216 (47.37)	
Middle school or above	193 (21.19)	78 (17.14)	115 (25.22)	
Alcohol consumption, n (%)				< 0.001
Never	557 (61.14)	310 (68.13)	247 (54.17)	
Former	68 (7.46)	22 (4.84)	46 (10.09)	
Current	286 (31.39)	123 (27.03)	163 (35.75)	
Smoking habits, n (%)				< 0.001
Never	588 (64.54)	342 (75.16)	246 (53.95)	
Former	141 (15.48)	43 (9.45)	98 (21.49)	
Current	182 (19.98)	70 (15.38)	112 (24.56)	
Body mass index (kg/m^2^)	25.11 (3.53)	24.87 (3.42)	25.35 (3.63)	0.04
Hypertension, n (%)	620 (68.06)	294 (64.62)	326 (71.49)	0.03
Diabetes, n (%)	137 (15.04)	64 (14.07)	73 (16.01)	0.4
Dyslipidemia, n (%)	223 (24.48)	116 (25.49)	107 (23.46)	0.5
Stroke, n (%)	104 (11.42)	35 (7.69)	69 (15.13)	< 0.001
Coronary heart disease, n (%)	165 (18.11)	79 (17.36)	86 (18.86)	0.6
Daily wear time, min	853.63 (79.53)	835.48 (73.95)	871.74 (80.85)	< 0.001
Wear seasons, n (%)				0.012
Spring	169 (18.55)	89 (19.56)	80 (17.54)	
Summer	417 (45.77)	189 (41.54)	228 (50)	
Autumn	246 (27)	142 (31.21)	104 (22.81)	
Winter	79 (8.67)	35 (7.69)	44 (9.65)	
% in sedentary behavior	57.18 (12.94)	47.79 (9.35)	66.54 (8.47)	< 0.001
% in LPA	35.02 (9.15)	41.22 (6.50)	28.83 (6.98)	< 0.001
% in MVPA	7.81 (5.93)	11.00 (6.31)	4.63 (3.22)	< 0.001

Data are mean (standard deviation), unless otherwise specified

*SD *standard deviation; *MRI *magnetic resonance imaging; *LPA *light-intensity physical activity; *MVPA *moderate-to-vigorous-intensity physical activity

Total Sedentary Time, median (Q1-Q3): 8.06 (6.81–9.37) hours. Low: ≤ 8.06 hours; High: >8.06 hours

### Sedentary behavior parameters and Gray matter volume of predefined brain regions

**Fig. 2:**
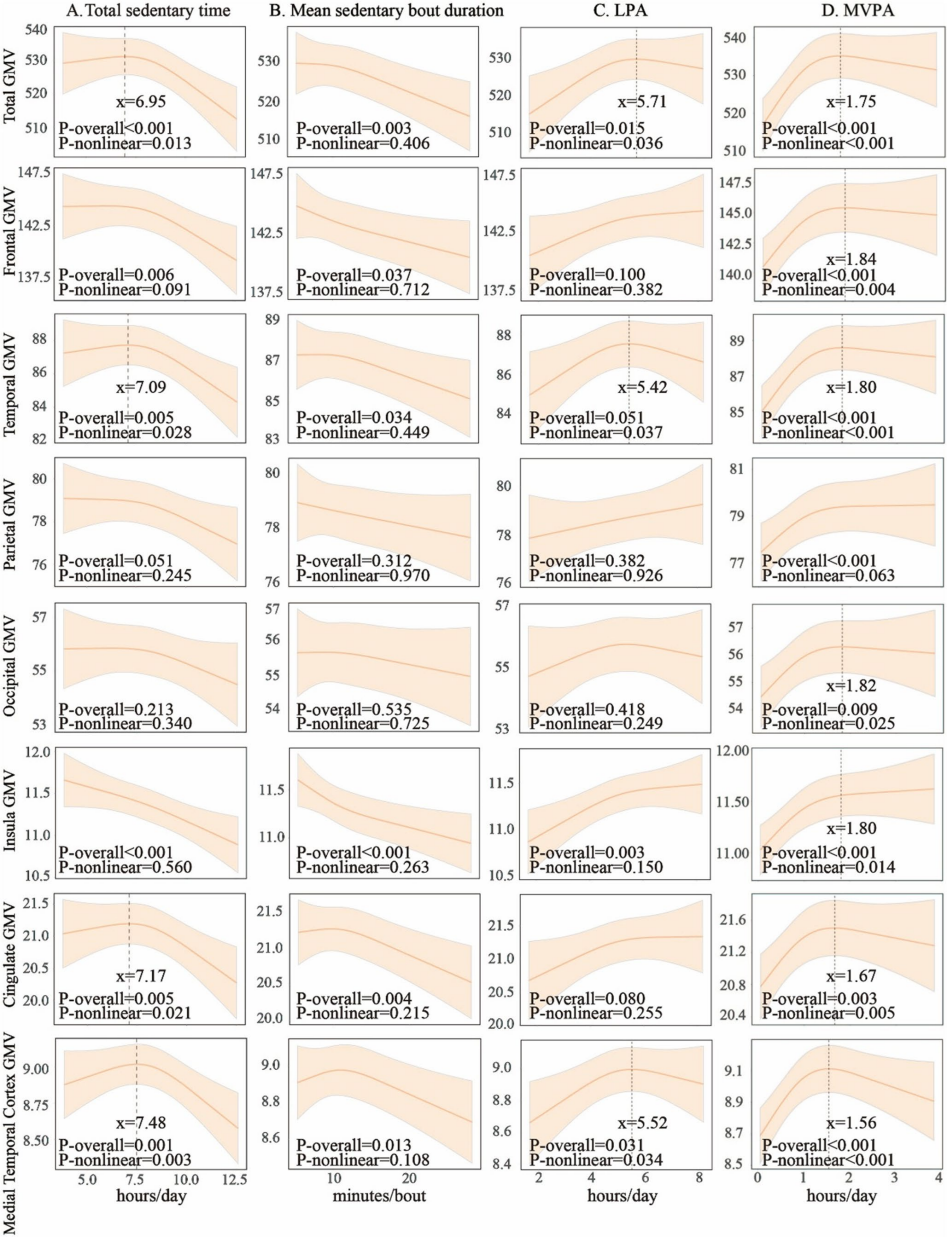
Association patterns of Total sedentary time (**A**), Mean sedentary bout duration (**B**), Light-intensity physical activity (**C**), and Moderate-to-vigorous physical activity (**D**) with gray matter volumes of predefined brain regions. Data were fitted using restricted cubic spline models. The solid lines and shaded areas represent the β coefficients and 95% confidence intervals of gray matter volumes (cm³) associated with sedentary behavior parameters. Models were adjusted for age, sex, education, wear time, wear season, intracranial volume, body mass index, smoking habits, alcohol intake, hypertension, diabetes, dyslipidemia, stroke, and coronary heart disease. The GMVs of each brain region are measured in cubic centimeters

Restricted cubic spline analysis indicated non-linear relationships of total sedentary time with GMVs in the total, temporal, cingulate cortex, and medial temporal cortex, with the inflection point (rounded to an integer) being ~ 7 h (all *p*-nonlinear < 0.05, Fig. 2A). Specifically, there was no significant association between total sedentary time and the GMV in the aforementioned brain regions on the left of inflection point, whereas on the right of inflection point (total sedentary time > 7 h), prolonged total sedentary time was linearly associated with lower GMVs in the aforementioned brain regions (all *p* < 0.05). We observed the Linear relationships of total sedentary time with frontal, parietal, and insula GMVs. Prolonged total sedentary time was significantly associated with lower frontal, parietal, and insula GMVs after adjusting for age, sex, education, ActiGraph wear time, wear season, and total ICV. These results remained largely the same after further adjusting for confounding factors in model 2 (Table 2). However, prolonged total sedentary time was Linearly correlated with lower insula GMV even after additionally adjusting for MVPA in model 3 (*p* < 0.05). In addition, longer mean sedentary bout duration was linearly associated with smaller GMVs of the total, frontal, temporal, insula, cingulate cortex, and medial temporal cortex (all *p*-nonlinear > 0.05, *p*-overall < 0.05, Fig. 2B).

**Table 2:** Association between sedentary behavior parameters and Gray matter volume of predefined brain regions (*n* = 911)

Sedentary parameters	β coefficient (95% confidence interval), GMV by brain regions		
	Model 1	Model 2	Model 3
Total GMV			
Total sedentary time ^a^			
> 7.0 h (*n* = 656)	**−5.14 (−7.10**,** −3.19)** ^**‡**^	**−4.56 (−6.55**,** −2.58)** ^**‡**^	**−3.12 (−5.64**,** −0.60)** ^*^
≤ 7.0 h (*n* = 255)	−1.13 (−5.20, 2.94)	−1.57 (−5.75, 2.60)	0.80 (−4.48, 6.09)
Mean sedentary bout duration (minutes) ^b^	**−0.81 (−1.19**,** −0.42)** ^**‡**^	**−0.66 (−1.05**,** −0.26)** ^**†**^	**−0.50 (−0.92**,** −0.08)** ^*^
LPA			
< 5.5 h (*n* = 589)	**5.21 (2.22**,** 8.21)** ^**†**^	**4.14 (1.10**,** 7.18)** ^**†**^	2.36 (−0.83, 5.55)
≥ 5.5 h (*n* = 322)	−1.59 (−5.91, 2.74)	−2.10 (−6.51, 2.31)	−2.13 (−6.57, 2.31)
MVPA			
< 1.8 h (*n* = 752)	**12.99 (7.90**,** 18.09)** ^**‡**^	**11.80 (6.64**,** 16.96)** ^**‡**^	
≥ 1.8 h (*n* = 159)	0.35 (−3.48, 4.19)	−2.85 (−8.60, 2.89)	
**Frontal GMV**			
Total sedentary time (hours) ^a^	**−0.65 (−1.06**,** −0.23)** ^**†**^	**−0.59 (−1.02**,** −0.16)** ^**†**^	−0.35 (−0.98, 0.27)
Mean sedentary bout duration (minutes) ^b^	**−0.20 (−0.33**,** −0.07)** ^**†**^	**−0.17 (−0.31**,** −0.04)** ^*^	−0.13 (−0.27, 0.02)
LPA (hours)	**0.66 (0.08**,** 1.24)** ^*^	**0.59 (0.0002**,** 1.18)** ^*^	0.35 (−0.27, 0.98)
MVPA			
< 1.8 h (*n* = 752)	**3.22 (1.50**,** 4.93)** ^**‡**^	**2.97 (1.22**,** 4.71)** ^**†**^	
≥ 1.8 h (*n* = 159)	0.14 (−1.27, 1.54)	−0.66 (−2.78, 1.46)	
**Temporal GMV**			
Total sedentary time ^a^			
> 7.0 h (*n* = 656)	**−0.98 (−1.41**,** −0.56)** ^**‡**^	**−0.86 (−1.29**,** −0.43)** ^**‡**^	−0.50 (−1.05, 0.05)
≤ 7.0 h (*n* = 255)	−0.12(−0.97, 0.74)	−0.24 (−1.13, 0.65)	0.52 (−0.60, 1.64)
Mean sedentary bout duration (minutes) ^b^	**−0.14 (−0.22**,** −0.05)** ^**†**^	**−0.11 (−0.19**,** −0.02)** ^*^	−0.07 (−0.16, 0.02)
LPA			
< 5.5 h (*n* = 589)	**0.92 (0.28**,** 1.56)** ^**†**^	**0.72 (0.07**,** 1.37)** ^*^	0.37 (−0.32, 1.05)
≥ 5.5 h (*n* = 322)	−0.54 (−1.48, 0.41)	−0.66 (−1.62, 0.30)	−0.71 (−1.67, 0.26)
MVPA			
< 1.8 h (*n* = 752)	**2.46 (1.36**,** 3.57)** ^**‡**^	**2.20 (1.07**,** 3.32)** ^**‡**^	
≥ 1.8 h (*n* = 159)	0.09 (−0.69, 0.87)	−0.51 (−1.67, 0.66)	
**Parietal GMV**			
Total sedentary time (hours) ^a^	**−0.32 (−0.54**,** −0.10)** ^**†**^	**−0.24 (−0.46**,** −0.02)** ^*^	−0.10 (−0.43, 0.22)
Mean sedentary bout duration (minutes) ^b^	**−0.08 (−0.15**,** −0.01)** ^*^	−0.05 (−0.12, 0.02)	−0.03 (−0.10, 0.05)
LPA (hours)	0.30 (−0.001, 0.60)	0.22 (−0.09, 0.52)	0.10 (−0.22, 0.43)
MVPA (hours)	**0.71 (0.26**,** 1.16)** ^**†**^	**0.56 (0.10**,** 1.02)** ^*^	
**Occipital GMV**			
Total sedentary time (hours) ^a^	−0.19 (−0.39, 0.01)	−0.15 (−0.36, 0.05)	0.01 (−0.28, 0.31)
Mean sedentary bout duration (minutes) ^b^	−0.05 (−0.11, 0.01)	−0.03 (−0.10, 0.03)	−0.01 (−0.08, 0.06)
LPA (hours)	0.13 (−0.15, 0.41)	0.09 (−0.19, 0.37)	−0.01 (−0.31, 0.28)
MVPA			
< 1.8 h (*n* = 752)	**1.39 (0.58**,** 2.20)** ^**†**^	**1.26 (0.43**,** 2.09)** ^**†**^	
≥ 1.8 h (*n* = 159)	−0.07 (−0.75, 0.62)	−0.48 (−1.52, 0.56)	
**Insula GMV**			
Total sedentary time (hours) ^a^	**−0.09 (−0.13**,** −0.04)** ^**‡**^	**−0.09 (−0.13**,** −0.04)** ^**‡**^	**−0.07 (−0.13**,** −0.002)** ^*^
Mean sedentary bout duration (minutes) ^b^	**−0.03 (−0.04**,** −0.01)** ^**‡**^	**−0.03 (−0.04**,** −0.01)** ^**‡**^	**−0.02 (−0.03**,** −0.004)** ^*^
LPA (hours)	**0.10 (0.04**,** 0.16)** ^**†**^	**0.10 (0.04**,** 0.15)** ^**†**^	**0.07 (0.002**,** 0.13)** ^*^
MVPA			
< 1.8 h (*n* = 752)	**0.31 (0.14**,** 0.49)** ^**†**^	**0.32 (0.15**,** 0.50)** ^**‡**^	
≥ 1.8 h (*n* = 159)	0.12(−0.02, 0.25)	0.01(−0.19, 0.21)	
**Cingulate Cortex GMV**			
Total sedentary time ^a^			
> 7.0 h (*n* = 656)	**−0.22 (−0.33**,** −0.11)** ^**‡**^	**−0.20 (−0.32**,** −0.09)** ^**‡**^	**−0.18 (−0.33**,** −0.04)** ^*^
≤ 7.0 h (*n* = 255)	−0.05 (−0.30, 0.20)	−0.08 (−0.34, 0.18)	0.01 (−0.32, 0.35)
Mean sedentary bout duration (minutes) ^b^	**−0.04 (−0.06**,** −0.02)** ^**‡**^	**−0.04 (−0.06**,** −0.01)** ^**†**^	**−0.03 (−0.06**,** −0.01)** ^**†**^
LPA (hours)	0.11 (0.01, 0.21) ^*^	0.10 (−0.001, 0.20)	0.07 (−0.03, 0.18)
MVPA			
< 1.8 h (*n* = 752)	**0.51 (0.22**,** 0.80)** ^**†**^	**0.48 (−0.18**,** 0.78)** ^**†**^	
≥ 1.8 h (*n* = 159)	0.12 (−0.11, 0.36)	−0.15 (−0.51, 0.21)	
**Medial Temporal Cortex GMV**			
Total sedentary time ^a^			
> 7.0 h (*n* = 656)	**−0.12 (−0.17**,** −0.06)** ^**‡**^	**−0.11 (−0.16**,** −0.06)** ^**‡**^	**−0.07 (−0.14**,** −0.01)** ^*^
≤ 7.0 h (*n* = 255)	0.003 (−0.09, 0.10)	−0.02 (−0.11, 0.08)	0.01 (−0.11, 0.13)
Mean sedentary bout duration (minutes) ^b^	**−0.01 (−0.02**,** −0.005)** ^**†**^	**−0.01 (−0.02**,** −0.003)** ^*^	−0.01 (−0.02, 0.0002)
LPA			
< 5.5 h (*n* = 589)	**0.10 (0.02**,** 0.17)** ^*^	**0.09 (0.01**,** 0.16)** ^*^	0.05 (−0.03, 0.13)
≥ 5.5 h (*n* = 322)	−0.07 (−0.18 0.04)	−0.08 (−0.19, 0.03)	−0.08 (−0.19, 0.04)
MVPA			
< 1.8 h (*n* = 752)	**0.28 (0.15**,** 0.41)** ^**‡**^	**0.27 (0.14**,** 0.40)** ^**‡**^	
≥ 1.8 h (*n* = 159)	0.01 (−0.08, 0.10)	−0.10 (−0.24, 0.04)	

*GMV *gray matter volume; *LPA *light-intensity physical activity (hours/day); *MVPA *moderate-to-vigorous-intensity physical activity (hours/day). ^a^ Total sedentary time was corrected for accelerometer wear-time and expressed as the estimated sedentary time (hours/day). ^b^ Mean sedentary bout Duration was the average length of time in all Sedentary bouts in minutes. Model 1: adjusted for age, sex, education, ActiGraph wear time and wear season, and total intracranial volume; Model 2: adjusted for the covariates in model 1 plus body mass index, smoking, alcohol intake, hypertension, diabetes, dyslipidemia, stroke, and coronary heart disease; and in Model 3 moderate-to-vigorous physical activity was added to model 2. ^*^P < 0.05, ^†^*P* < 0.01, ^‡^*P* < 0.001

For the relationships of LPA and MVPA with GMVs in predefined brain regions, restricted cubic spline analysis showed a linear relationship between LPA time and insula GMV (*p*-nonlinear > 0.05, *p*-overall < 0.05, Fig. 2C). We also identified non-linear relationships of LPA with GMVs in certain regions such that LPA was not significantly associated with the regional GMVs on the right of inflection point (LPA ≥ 5.5 h), whereas on the left of inflection point, more time spent in LPA was significantly associated with increased total, temporal and medial temporal cortex GMV. MVPA time exhibited significant non-linear relationships with GMVs in the total brain, frontal, temporal, occipital, insula, cingulate, and medial temporal cortex, but a significant linear association with parietal GMV (*p*-nonlinear < 0.05, Fig. 2D).

To provide a more comprehensive understanding of the relationship between sedentary behavior metrics and regional GMVs, we further explored the associations of prolonged sedentary time, the number of breaks per sedentary hour, usual sedentary bout duration, and alpha with GMVs in the predefined brain regions (Supplementary Table 1). Prolonged sedentary time was significantly associated with smaller GMVs in the total, frontal, temporal, insula, and cingulate cortex (all *p* < 0.05). Beyond the inflection point of prolonged sedentary time (> 3.35 h), more time spent in sedentary behavior was significantly associated with lower GMVs in the medial temporal cortex. Additionally, a prolonged sedentary bout duration was significantly associated with smaller GMVs in the total brain, insula, and cingulate cortex (all *p*-nonlinear > 0.05, *p*-overall < 0.05, Fig. S1C). The number of breaks per sedentary hour showed significant non-linear associations with GMVs in the total brain, temporal, cingulate, and medial temporal cortex (*p*-nonlinear < 0.05, Fig. S1B). Moreover, an increased number of breaks per sedentary hour was significantly associated with higher GMVs in the insula (*p* < 0.05). Finally, alpha showed significant non-linear associations with GMVs in the total brain, temporal, insula, cingulate, and medial temporal cortex (all *p*-nonlinear > 0.05, *p*-overall < 0.05, Fig. S1D).

### Sedentary behavior parameters and gray matter atrophy patterns

**Fig. 3:**
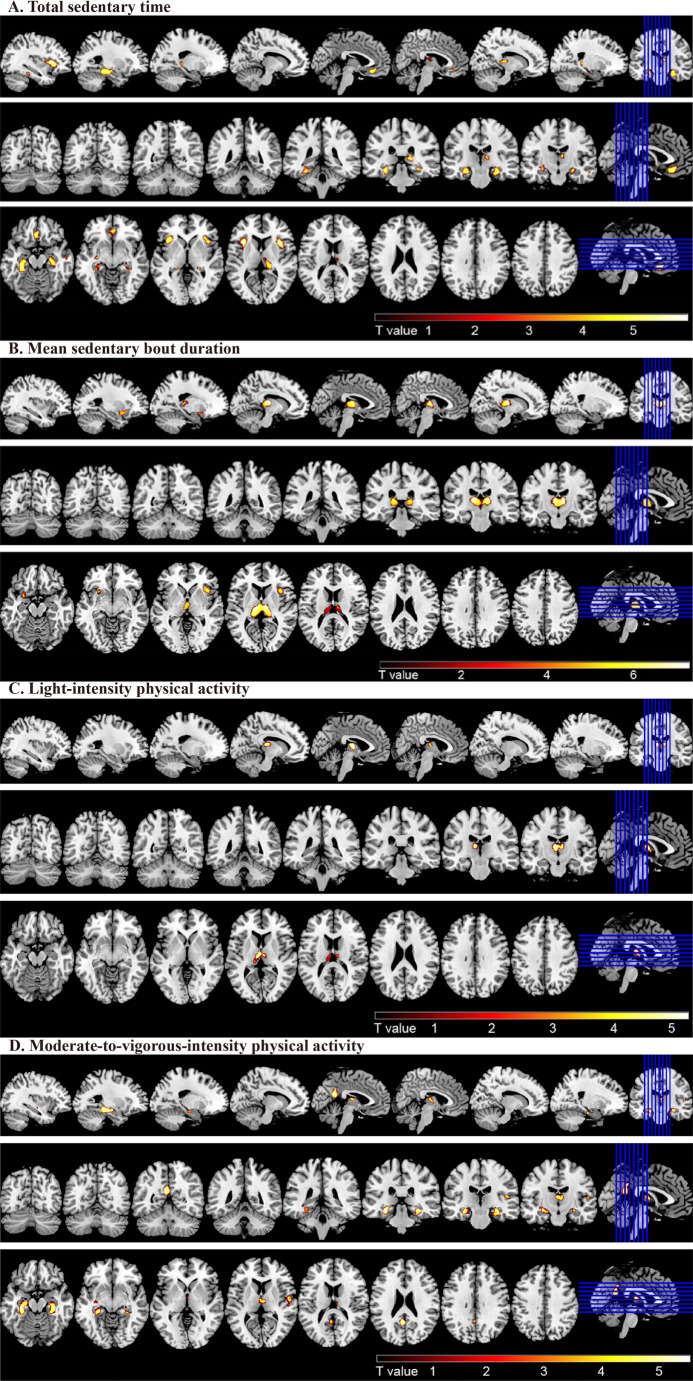
Gray matter regions that were correlated with total sedentary time (**A**), the mean sedentary bout duration (**B**), Light-intensity physical activity (**C**) and Moderate-to-vigorous-intensity physical activity (**D**). **A.** The brain regions of reduced gray matter volume associated with total sedentary time mainly involved the left hippocampus, left insula, right inferior frontal operculum, right hippocampus, left medial orbitofrontal cortex and right middle temporal gyrus. **B.** The brain regions of reduced gray matter volume associated with mean sedentary bout duration included left thalamus, right inferior frontal operculum and left insula. **C.** The brain regions showing increased gray matter volume associated with light-intensity physical activity were primarily located in the left thalamus. **D.** The brain regions of increased gray matter volume associated with moderate-to-vigorous physical activity primarily included the left hippocampus, right hippocampus, left precuneus, right thalamus and right superior temporal gyrus. The analyses were adjusted for age, sex, education, ActiGraph wear time and wear season, total intracranial volume, smoking habits, alcohol intake, and body mass index.

VBM analysis showed that greater daily total sedentary time was significantly correlated with smaller volumes of the left hippocampus, left insula, right inferior frontal operculum, right hippocampus, left medial orbitofrontal cortex, and right middle temporal gyrus in people who spent more than 7 h per day in sedentary behavior (Fig. 3). Table 3 shows MNI coordinates for this analysis. In addition, the longer mean sedentary bout Duration was correlated with smaller volumes of the left thalamus, right opercular part of the inferior frontal gyrus, and left insula. Besides, the brain regions showing increased GMVs associated with LPA were primarily located in the left thalamus in individuals who engaged in less than 5.5 h per day in LPA. In individuals who engaged in less than 1.8 h per day in MVPA, the brain regions with increased GMVs associated with MVPA primarily included the left hippocampus, right hippocampus, left precuneus, right thalamus, and right superior temporal gyrus.

**Table 3:** Summary of brain regions that showed significant associations of grey matter volume with total sedentary time and mean sedentary bout duration

Brain regions	MNI coordinates			Cluster size (voxels)	z-score	pos./neg.	*P*-value^*^	Peak T value
	x	y	z					
Total sedentary time (hours) (*n* = 656)								
L Hippocampus	−33	−33	−12	764	5.40	neg.	< 0.001	5.47
L Insula	−35	17	8	668	5.93	neg.	< 0.001	6.01
R Inferior Frontal Operculum	39	15	6	611	5.87	neg.	< 0.001	5.95
R Hippocampus	35	−24	−17	515	5.02	neg.	0.002	5.07
L Medial Orbitofrontal Cortex	2	33	−14	396	5.27	neg.	0.001	5.32
R Hippocampus	17	−35	8	327	4.92	neg.	0.003	4.96
R Middle Temporal Gyrus	54	2	−27	251	4.97	neg.	0.003	5.02
**Mean sedentary bout duration (minutes)** (*n* = 911)								
L Thalamus	−11	−18	12	2134	7.24	neg.	< 0.001	7.34
R Inferior Frontal Operculum	39	17	3	482	6.03	neg.	< 0.001	6.09
L Insula	−23	14	−12	259	5.18	neg.	0.001	5.22
**LPA (hours)** (*n* = 589)								
L Thalamus	−5	−12	12	549	5.20	pos	0.001	5.26
**MVPA (hours)**(*n* = 752)								
L Hippocampus	−30	−30	−14	681	5.54	pos	< 0.001	5.60
R Hippocampus	33	−32	−11	587	5.50	pos	< 0.001	5.56
L Precuneus	−5	−54	23	355	5.35	pos	0.001	5.40
R Thalamus	3	−14	12	226	4.68	pos	0.05	4.68
R Superior Temporal Gyrus	56	−3	5	214	4.79	pos	0.05	4.83

*MNI*, Montreal Neurological Institute; *L *left; *R *right; *LPA *light-intensity physical activity; *MVPA *moderate-to-vigorous-intensity physical activity

^*^P-values corrected for multiple comparisons by controlling the family-wise error across the whole brain, and using extent threshold of > 200 voxels

Values were adjusted for age, sex, education, ActiGraph wear time and wear season, total intracranial volume, smoking habits, alcohol intake, and body mass index

Prolonged sedentary time was significantly correlated with a smaller volume of the thalamus (Supplementary Table 2). The prolonged usual sedentary bout duration was significantly correlated with smaller volumes in the right thalamus and the right inferior frontal gyrus and opercular part. Additionally, alpha was significantly associated with greater GMVs in the left thalamus, insula, left putamen, right middle temporal gyrus, right medial orbital part of the frontal lobe, and left fusiform gyrus. The number of breaks per sedentary hour was not significantly associated with the GMVs in any of the examined brain regions after controlling for family-wise error across the entire brain, using an extent threshold of > 200 voxels.

## Discussion

This population-based neuroimaging study of older adults showed consistent associations between sedentary behavior patterns and reduced GMVs in multiple brain regions, particularly those regions related to cognitive and emotional regulation. Specifically, total sedentary time exceeding 7 h/day was associated with reduced GMVs in the hippocampus, temporal, frontal, cingulate, and insula regions. Furthermore, prolonged mean and usual sedentary bout durations showed similarly widespread associations with reduced GMVs in the thalamus and frontal opercular cortex. In addition, our study further demonstrated that breaking up sedentary time more frequently (i.e., a higher number of breaks per sedentary hour) was associated with larger GMVs, particularly in the insula, the finding that aligns with the “breaker” hypothesis. Finally, we observed that greater time spent in LPA and MVPA was associated with enlarged GMVs in brain regions such as the thalamus and hippocampus. Taken together, these findings support the notion that both the total sedentary time and the patterns of sedentary behavior accumulation are critical for brain health in aging.

Population-based studies have rarely explored the association between objectively-measured sedentary behavior parameters and regional GMVs among older adults. Our community-based study revealed the daily sedentary time-dependent relationships with GMVs in total, temporal, cingulate, and medial temporal cortex among rural-dwelling dementia-free older adults. Specifically, GMVs in these brain regions were not related with daily sedentary behavior time up to around 7 h of sedentary time, but thereafter GMVs was linearly decreased with increasing daily sedentary behavior time. This is in line with the report from a large-scale cohort study in Japan suggesting that prolonged daily sedentary behavior is associated with increased risks of dementia and functional disability (Du et al., 2024). Indeed, as individuals age, the daily time spent in sedentary behavior gradually increases, primarily due to age-related declines in the functional capacity of the cardiorespiratory and muscular systems (Izquierdo et al., 2021; Lavie et al., 2015). Nevertheless, given the cross-sectional nature of our study, further prospective cohort studies are warranted to investigate the temporal relationships of sedentary behavior parameters with global and regional gray matter atrophy across ethnically and socioculturally diverse populations.

Another important finding from our study was that not only total sedentary time, but also certain patterns of sedentary behavior was associated with structural brain alterations. Previous studies have consistently linked prolonged sedentary time (a sedentary metric that is highly correlated with prolonged sedentary bout duration and infrequent breaks in sedentary behavior) with cardiometabolic risk factors, cardiovascular disease, and mortality (Bellettiere et al., 2019; Diaz et al., 2017b; Healy et al., 2011). These findings highlight the significance of the “prolonger” versus “breaker” hypothesis, emphasizing that both the quantity of sedentary time and the way it is accumulated play crucial roles in brain and cognitive health (Dunstan et al., 2021). Our study revealed that longer mean sedentary bout duration was associated with smaller GMVs in total, frontal, temporal, insula, cingulate, and medial temporal cortex and that more time spent in LPA and MVPA was linked to greater insula GMV.

Our VBM analysis revealed that prolonged total sedentary time was correlated with atrophy in the left hippocampus, left insula, right inferior frontal operculum, right hippocampus, left medial orbitofrontal cortex, and right middle temporal gyrus, which represents the third contribution of our study to the current literature. In addition, increased mean sedentary bout duration was correlated with atrophy in the left thalamus, right opercular part of the inferior frontal gyrus, and left insula. Daily LPA time was associated with increased GMVs in the left thalamus, while MVPA time was mainly linked to larger GMVs in the right thalamus. The insula serves as a central brain hub with extensive connections and diverse functional roles. Atrophy in the insula has been implicated in various neurodegenerative diseases such as frontotemporal dementia (FTD), Alzheimer’s disease (AD), and Parkinson’s disease (PD)(Fathy et al., 2020). The thalamus is a crucial node within networks that supports function of multiple cognitive domains, such memory, executive function, attention, and information processing (Chen et al., 2017; Fama & Sullivan, 2015). Previous studies have reported that the accumulation patterns of prolonged sedentary time were linearly associated with poorer global cognition, memory, and verbal fluency function, in which association was predominantly mediated by structural brain lesions (Han et al., 2023). The atrophy observed in brain regions associated with sedentary behavior patterns in our study may contribute to accelerated decline in functions of these brain regions.

The mechanisms linking sedentary time and accumulation patterns with gray matter atrophy are not fully understood. Uninterrupted long sitting time has been linked with a significant decline in blood flow velocity in the middle cerebral artery (Carter et al., 2018). Furthermore, longer sedentary time was associated with reduced cerebral blood flow in lateral and medial frontal regions (Zlatar et al., 2019), which in turn, may damage brain structure and lead to cognitive dysfunction (Hakim, 2021).

This is a large-scale, population-based study that engaged dementia-free older adults who were living in rural communities in north China and a considerable proportion of the participants received no or very limited formal education and had low socioeconomic status. This demographic group has historically been significantly underrepresented in research on lifestyles, movement behaviors, and brain health (Lock et al., 2023). Our study does have limitations. Accelerometers cannot entirely distinguish activities between sitting and standing postures, and thus, activities in both postures could be classified as sedentary behaviors. Furthermore, our cross-sectional design does not allow us to infer a causal relationship for any of the observed associations between sedentary behavior parameters and patterns of gray matter atrophy. Finally, participants in MIND-China were derived from only one rural area in western Shandong province and those in the ActiGraph and MRI substudies were relatively younger and healthier compared with the MIND-China target population. This should be kept in mind when generalizing the research findings to broader populations.

## Conclusion

Our population-based study provides comprehensive evidence that both the total sedentary time and the patterns of sedentary behavior are associated with GMVs in key brain regions of older adults. Notably, prolonged total sedentary time and longer uninterrupted sitting periods were associated with extensive gray matter atrophy, particularly in brain regions being involved in memory, executive function, and emotional regulation. Moreover, our study supports the “breaker” hypothesis that more frequent breaks in sedentary behavior were associated with greater GMVs in several critical brain regions. In parallel, greater daily time spent in LPA and MVPA was also related to larger GMVs in brain regions such as the thalamus and hippocampus. These findings suggest that both increasing physical activity and frequently interrupting sedentary time may have the potential to preserve brain structure and reduce the risk of neurodegeneration. Future longitudinal and interventional studies are warranted to confirm these associations, which may facilitate behavioral interventions to promote brain health in aging populations.

## Supplementary Information

Below is the link to the electronic supplementary material.

Supplementary File 3 (DOCX 1.61 MB)

## Data Availability

All the data used to support our findings are available from the corresponding authors upon reasonable request and approval by the MIND-China Steering Committee.
