# Supplementary material for: Characterizing Gray matter atrophy patterns associated with accelerometer-measured sedentary behavior: a population-based study
Source: Brain Imaging Behav. 2025 Sep 30;19(6):1379–93. doi: 10.1007/s11682-025-01054-1 (PMC12831676; doi:10.1007/s11682-025-01054-1)
Supplement: Supplementary file 1 — Supplementary File 3 (DOCX 1.61 MB) [file 11682_2025_1054_MOESM1_ESM.docx]

**Supplementary materials**

**Characterizing gray matter atrophy patterns associated with accelerometer-measured sedentary behavior: a population-based study**

Tian M, et al.

**Methods**

**Data collection, assessments, and definitions**

In March-September 2018, trained medical staff collected extensive data following a structured questionnaire, which included sociodemographic characteristics (e.g., age, sex, and education), behavioral factors (e.g., smoking, alcohol consumption, and physical activity), metabolic factors (e.g., hypertension, diabetes, dyslipidemia, and cardiovascular disease), use of medications in the two weeks prior to the survey (e.g., antihypertensives, blood glucose-lowering, and lipid-lowering drugs), blood tests (e.g., blood glucose and lipids), and clinical conditions (e.g., sinonasal disease)(Han et al., 2021; Wang et al., 2022). Weight and height were measured with participants wearing light clothing and no shoes. BMI was calculated as weight (kg) divided by height squared (m²). After a 5-minute rest, arterial blood pressure was measured on the right upper arm in a seated position using an electronic blood pressure monitor (HEM-7127J, Omron Corporation, Kyoto, Japan).

Hypertension was defined as systolic pressure ≥140 mmHg or diastolic pressure ≥90 mmHg or current use of antihypertensive medication(Lu et al., 2017). High cholesterol was defined as a total cholesterol ≥6.22 mmol/L or receiving treatment for high cholesterol(Song et al., 2019). Diabetes was defined as a self-reported history of diabetes diagnosed by a physician or fasting blood glucose ≥7.0 mmol/L or current use of blood glucose-lowering medication(Liu et al., 2016).

**Definitions of sedentary behavior parameters**

All metrics related to sedentary behavior were evaluated based on the widely used measure of sedentary time, defined as having <100 counts per minute recorded on the vertical axis(Han et al., 2022). Total sedentary time was defined as the average time (in minutes) spent in sedentary bout for ≥1 minute per day. A sedentary bout was defined as a consecutive period of time (in minutes) recorded on the accelerometer during which the counts per minute were <100. The mean sedentary bout duration was calculated using data from all valid days by dividing the total sedentary time (in minutes) by the total number of sedentary bouts. Lower bout durations suggest interrupted patterns, whereas higher bout durations indicate more continuous accumulation patterns. We also assessed the prolonged sedentary time (time spent in sedentary bouts ≥30 min)(Han et al., 2023), the number of breaks per sedentary hour was calculated as the total number of sedentary breaks per day divided by the total sedentary time in hours. This standardization ensures that the metric accurately reflects the frequency of interruptions relative to time spent in sedentary behavior, in support of the "breaker hypothesis" (Han et al., 2023). The usual sedentary bout duration represents the midpoint of the cumulative distribution of sedentary bout durations(de Rezende et al., 2014; Dempsey et al., 2022), and alpha is a unitless measure ranging from 1.3 to 2.1(Chastin & Granat, 2010).

**Structural MRI data processing**

We used the default preprocessing pipelines to quantify gray matter volume (GMV) as previously described, which included: (i) All the 3D T1-weighted images underwent bias-field inhomogeneity correction; (ii) The brain images were segmented into gray matter, white matter, and cerebrospinal fluid (CSF); (iii) A customized, study-specific template was generated using the Diffeomorphic Anatomical Registration using Exponentiated Lie algebra (DARTEL) algorithm; (iv) Gray matter density images were warped to the customized template and affine registered to the Montreal Neurological Institute (MNI) space; (v) The GMV map was derived by multiplying the gray matter density map with the nonlinear determinants obtained from spatial normalization; and (vi) The resulting GMV images were smoothed using a 6-mm full-width-at-half-maximum (FWHM) kernel(Song et al., 2023).

**Supplementary Table 1. Association between sedentary behavior parameters and gray matter volume of predefined brain regions.**

| **Sedentary parameters** | **β coefficient (95% confidence interval), regional GMV** | | |
| --- | --- | --- | --- |
|  | **Model 1** | **Model 2** | **Model 3** |
| **Total GMV** |  |  |  |
| Prolonged sedentary time (hours) | **-2.22 (-3.33, -1.10) ^‡^** | **-1.79 (-2.92, -0.66) ^†^** | -1.17 (-2.49, 0.15) |
| No. of breaks per sedentary hour | | | |
| >7.0 (n=332) | -0.02 (-2.08, 2.04) | -0.15 (-2.30 1.99) | -0.76 (-3.10, 1.59) |
| ≤7.0 (n=579) | **4.16 (1.93, 6.38) ^‡^** | **3.25 (0.99, 5.52) ^†^** | 1.93 (-0.50, 4.35) |
| Usual sedentary bout duration (minutes) | **-0.26 (-0.43, -0.10) ^†^** | **-0.21 (-0.37, -0.04) ^*^** | -0.14 (-0.31, 0.03) |
| Alpha |  |  |  |
| > 1.7 (n=215) | 20.59 (-33.94, 75.11) | 20.12 (-34.95, 75.20) | 7.63 (-51.35, 66.60) |
| ≤ 1.7 (n=696) | **79.12 (46.61, 111.65) ^‡^** | **67.49 (34.26, 100.71) ^‡^** | **44.76 (7.84, 81.67) ^*^** |
| **Frontal GMV** |  |  |  |
| Prolonged sedentary time (hours) | **-0.57 (-0.94, -0.20) ^†^** | **-0.53 (-0.92, -0.14) ^†^** | -0.35 (-0.80, 0.10) |
| No. of breaks per sedentary hour | **0.42 (0.08, 0.75) ^*^** | **0.36 (0.02, 0.70) ^*^** | 0.15 (-0.25, 0.56) |
| Usual sedentary bout duration (minutes) | **-0.07 (-0.13, -0.02) ^*^** | **-0.06 (-0.12, -0.01) ^*^** | -0.04 (-0.10, 0.01) |
| Alpha | **8.38 (1.89, 14.87) ^*^** | **7.43 (0.78, 14.09) ^*^** | 3.15 (-5.13, 11.43) |
| **Temporal GMV** |  |  |  |
| Prolonged sedentary time (hours) | **-0.37 (-0.61, -0.13) ^†^** | **-0.29 (-0.54, -0.05) ^*^** | -0.13 (-0.41, 0.16) |
| No. of breaks per sedentary hour | | | |
| > 7.0 (n=332) | -0.09 (-0.53, 0.35) | -0.16 (-0.62 0.30) | -0.32 (-0.82, 0.18) |
| ≤ 7.0 (n=579) | **0.75 (0.27, 1.23) ^†^** | **0.57 (0.08, 1.06) ^*^** | 0.28 (-0.24, 0.80) |
| Usual sedentary bout duration (minutes) | **-0.04 (-0.08, -0.01) ^*^** | -0.03 (-0.07, 0.004) | -0.02 (-0.05, 0.02) |
| Alpha |  |  |  |
| > 1.7 (n=215) | 5.23 (-6.19, 16.65) | 5.32 (-6.30, 16.93) | 0.79 (-11.57, 13.14) |
| ≤ 1.7 (n=696) | **15.99 (8.97, 23.01) ^‡^** | **13.41 (6.25, 20.56) ^‡^** | **8.59 (0.63, 16.54) ^*^** |
| **Parietal GMV** |  |  |  |
| Prolonged sedentary time (hours) | **-0.24 (-0.43, -0.04) ^*^** | -0.17 (-0.37, 0.03) | -0.06 (-0.29, 0.18) |
| No. of breaks per sedentary hour | 0.15 (-0.02, 0.33) | 0.09 (-0.09, 0.27) | -0.04 (-0.25, 0.17) |
| Usual sedentary bout duration (minutes) | -0.02 (-0.05, 0.01) | -0.01 (-0.04, 0.02) | -0.001 (-0.03, 0.03) |
| Alpha | **3.76 (0.37, 7.14) ^*^** | 2.48 (-0.98, 5.93) | -0.01 (-4.32, 4.29) |
| **Occipital GMV** |  |  |  |
| Prolonged sedentary time (hours) | -0.17, (-0.35, 0.01) | -0.13 (-0.31, 0.05) | -0.04 (-0.25, 0.17) |
| No. of breaks per sedentary hour | 0.11 (-0.06, 0.27) | 0.07 (-0.10, 0.23) | -0.04 (-0.23, 0.16) |
| Usual sedentary bout duration (minutes) | -0.01 (-0.04, 0.01) | -0.01 (-0.10, 0.23) | -0.003 (-0.03, 0.03) |
| Alpha | 2.35 (-0.74, 5.45) | 1.63 (-1.53, 4.79) | -0.55 (-4.49, 3.38) |
| **Insula GMV** |  |  |  |
| Prolonged sedentary time (hours) | **-0.08 (-0.12, -0.04) ^‡^** | **-0.08 (-0.12, -0.04) ^‡^** | **-0.06 (-0.10, -0.01) ^*^** |
| No. of breaks per sedentary hour | **0.06 (0.02, 0.09) ^†^** | **0.06 (0.02, 0.09) ^†^** | 0.03 (-0.01, 0.07) |
| Usual sedentary bout duration (minutes) | **-0.01 (-0.02, -0.004) ^†^** | **-0.01 (-0.02, -0.004) ^†^** | **-0.01 (-0.01, -0.001) ^*^** |
| Alpha |  |  |  |
| > 1.7 (n=215) | 1.10 (-0.64, 2.84) | 1.27 (-0.44, 2.97) | 0.65 (-1.17, 2.46) |
| ≤ 1.7 (n=696) | **2.46 (1.32, 3.60) ^‡^** | **2.41 (1.24, 3.58) ^‡^** | **2.00 (0.70, 3.30) ^†^** |
| **Cingulate Cortex GMV** |  |  |  |
| Prolonged sedentary time (hours) | **-0.11 (-0.17, -0.04) ^†^** | **-0.10 (-0.16, -0.03) ^†^** | **-0.09 (-0.16, -0.01) ^*^** |
| No. of breaks per sedentary hour | | | |
| > 7.0 (n=332) | 0.00 (-0.12, 0.13) | 0.01 (-0.13 0.14) | -0.01 (-0.16, 0.13) |
| ≤ 7.0 (n=579) | **0.20 (0.08, 0.32) ^†^** | **0.17 (0.05, 0.30) ^†^** | 0.13 (0.00, 0.27) |
| Usual sedentary bout duration (minutes) | **-0.02 (-0.02, -0.01) ^†^** | **-0.01 (-0.02, -0.004) ^†^** | **-0.01 (-0.02, -0.002) ^*^** |
| Alpha |  |  |  |
| > 1.7 (n=215) | 5.23 (-6.19, 16.65) | 5.32 (-6.30, 16.93) | 0.79 (-11.57, 13.14) |
| ≤ 1.7 (n=696) | **15.99 (8.97, 23.01) ^‡^** | **13.41 (6.25, 20.56) ^‡^** | **8.59 (0.63, 16.54) ^*^** |
| **Medial Temporal Cortex GMV** |  |  |  |
| Prolonged sedentary time |  |  |  |
| > 3.4 hours (n=495) | **-0.08 (-0.12, -0.03) ^†^** | **-0.08 (-0.12, -0.03) ^†^** | **-0.05 (-0.10, 0.00) ^*^** |
| ≤ 3.4 hours (n=416) | 0.00 (-0.10, 0.10) | 0.00 (-0.10, 0.11) | 0.01 (-0.11, 0.12) |
| No. of breaks per sedentary hour | | | |
| > 7.0 (n=332) | -0.03 (-0.08, 0.02) | -0.03 (-0.09, 0.02) | -0.04 (-0.10, 0.02) |
| ≤ 7.0 (n=579) | **0.08 (0.02, 0.14) ^†^** | **0.07 (0.01, 0.13) ^*^** | 0.04 (-0.02, 0.10) |
| Usual sedentary bout duration (minutes) | **-0.005 (-0.01, -0.001) ^*^** | -0.004 (-0.01, 0.0002) | -0.003 (-0.01, 0.001) |
| Alpha |  |  |  |
| > 1.7 (n=215) | 0.07 (-1.22, 1.36) | 0.08 (-1.26, 1.42) | 0.24 (-1.67, 1.20) |
| ≤ 1.7 (n=696) | **1.83 (0.99, 2.66) ^‡^** | **1.71 (0.86, 2.55) ^‡^** | **1.25 (0.31, 2.20) ^†^** |

Abbreviations: GMV, gray matter volume. Model 1: adjusted for age, sex, education, ActiGraph wear time and wear season, and total intracranial volume; Model 2: adjusted for the covariates in model 1 plus body mass index, smoking, alcohol intake, hypertension, diabetes, dyslipidemia, stroke, and coronary heart disease; and in Model 3 moderate-to-vigorous physical activity was added to model 2. ^*^*P*<0.05, ^†^*P*<0.01, ^‡^*P*<0.001.

| **Brain regions** | **MNI coordinates** | | | **Cluster size (voxels)** | **z-score** | **pos./neg.** | ***P*-value^*^** | **Peak T value** |
| --- | --- | --- | --- | --- | --- | --- | --- | --- |
|  | **x** | **y** | **z** |  |  |  |  |  |
| **Prolonged sedentary time (hours) (n=495)** |  |  |  |  |  |  |  |  |
| R Thalamus | 9 | -20 | 8 | 634 | 5.04 | neg. | 0.002 | 5.11 |
| L Thalamus | -8 | -17 | 11 | 212 | 4.57 | neg. | 0.015 | 4.63 |
| **Usual sedentary bout duration (minutes) ^a^ (n=911)** |  |  |  |  |  |  |  |  |
| R Thalamus | 9 | -14 | 9 | 1911 | 6.46 | neg. | <0.001 | 6.54 |
| R Inferior frontal gyrus, opercular part | 39 | 17 | 3 | 369 | 5.95 | neg. | <0.001 | 6.01 |
| **Alpha ^b^ (n=696)** |  |  |  |  |  |  |  |  |
| L Thalamus | -14 | -27 | 12 | 1134 | 5.20 | pos. | 0.001 | 5.26 |
| R Insula | 38 | 17 | 5 | 644 | 6.06 | pos. | <0.001 | 6.14 |
| L Putamen | -30 | -14 | -9 | 567 | 5.45 | pos. | <0.001 | 5.51 |
| R Middle Temporal Gyrus | 53 | 2 | -27 | 452 | 5.41 | pos. | <0.001 | 5.47 |
| R Medial Orbital Part of the Frontal Lobe | 5 | 38 | -11 | 387 | 5.25 | pos. | 0.001 | 5.31 |
| L Insula | -36 | 5 | 8 | 365 | 5.67 | pos. | <0.001 | 5.74 |
| L Fusiform Gyrus | -27 | -33 | -17 | 227 | 4.70 | pos. | 0.009 | 4.74 |

**Supplementary Table 2. A summary of brain regions that showed significant correlations of grey matter volumes with prolonged sedentary time, number of breaks per sedentary hour, alpha and usual sedentary bout duration.**

Abbreviation: MNI, Montreal Neurological Institute; L, left; R, right.

^*^*P*-values were corrected for multiple comparisons by controlling for the family-wise error across the whole brain, and using extent threshold of >200 voxels.

^a^ Usual sedentary bout duration (also known as the weighted median statistic w50 or x50) is the midpoint of the cumulative distribution of sedentary bout durations. Half of all sedentary behavior time is accumulated in bouts longer than the usual sedentary bout duration.

^b^ Alpha is a unitless measure ranging from 1.3 to 2.1 that characterizes the frequency distribution of sedentary bout durations. Higher values indicate sedentary behavior accumulation patterns with relatively shorter bouts (i.e., more frequently interrupted) and relatively fewer short bouts.

P-values were adjusted for age, sex, education, ActiGraph wear time and wear season, total intracranial volume, smoking, alcohol intake, and body mass index.


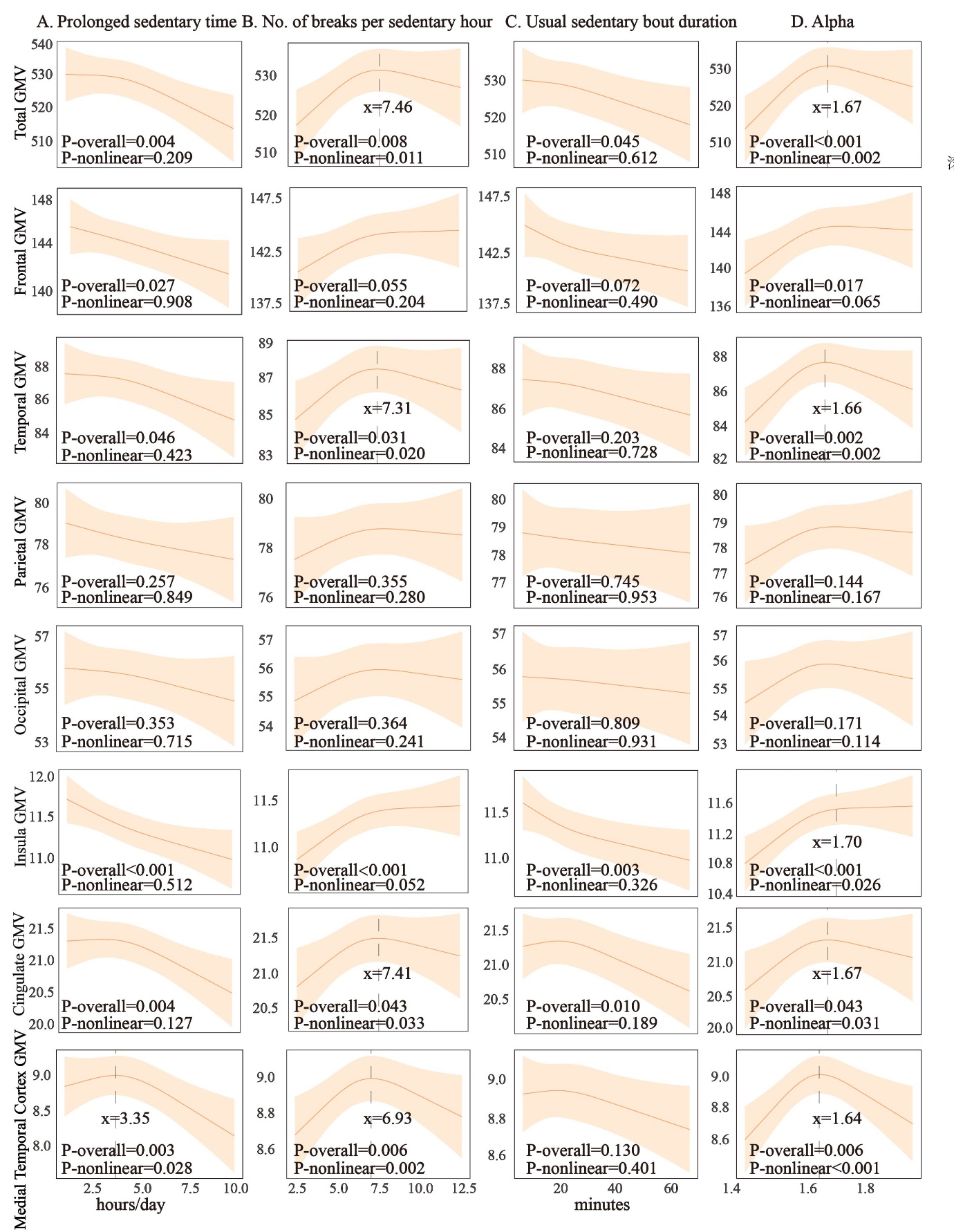


**Figure S1.** **Association patterns of prolonged sedentary time (A), number of breaks per sedentary hour (B), usual sedentary bout duration (C), and Alpha (D) with gray matter volume of predefined brain regions.**

Data were fitted using restricted cubic spline models. The solid lines and shaded areas represent the β coefficients and 95% confidence intervals of gray matter volumes (cm³), respectively. Models were adjusted for age, sex, education, wear time, wear season, intracranial volume, BMI, smoking habits, alcohol intake, hypertension, diabetes, dyslipidemia, stroke, and coronary heart disease.

**A. Prolonged sedentary time**

**
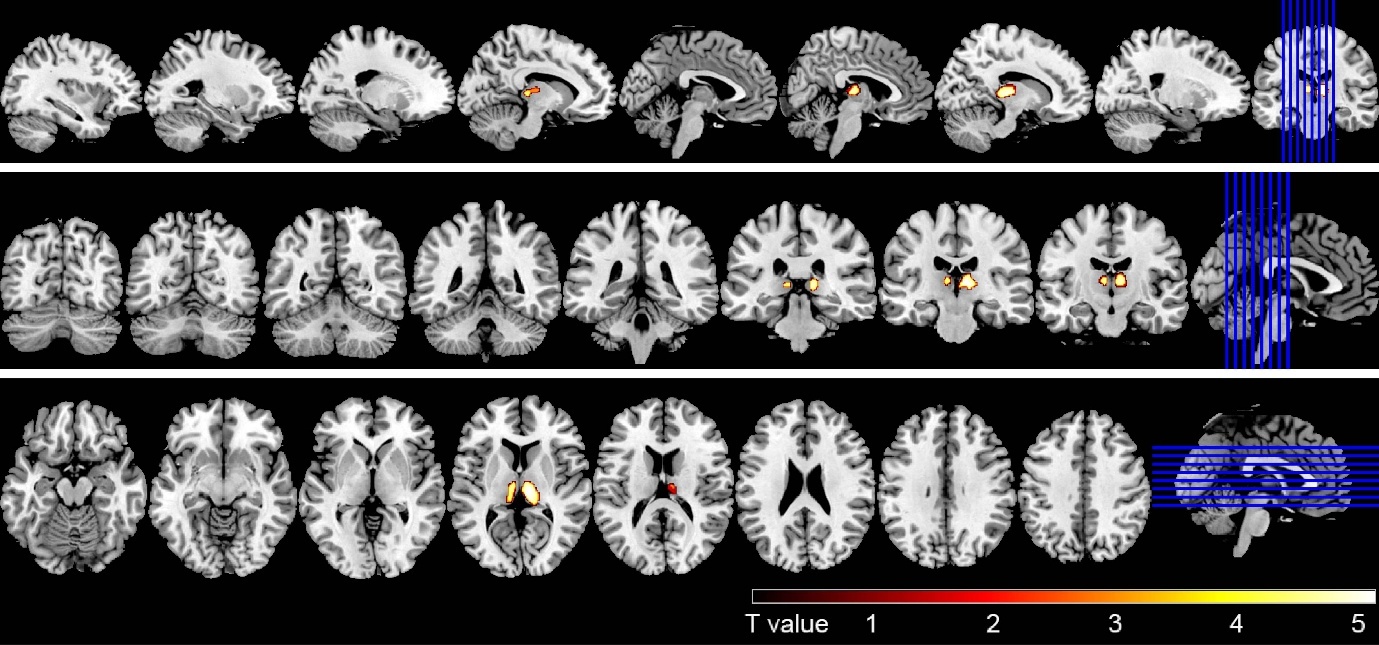
**

**B. Usual sedentary bout duration**


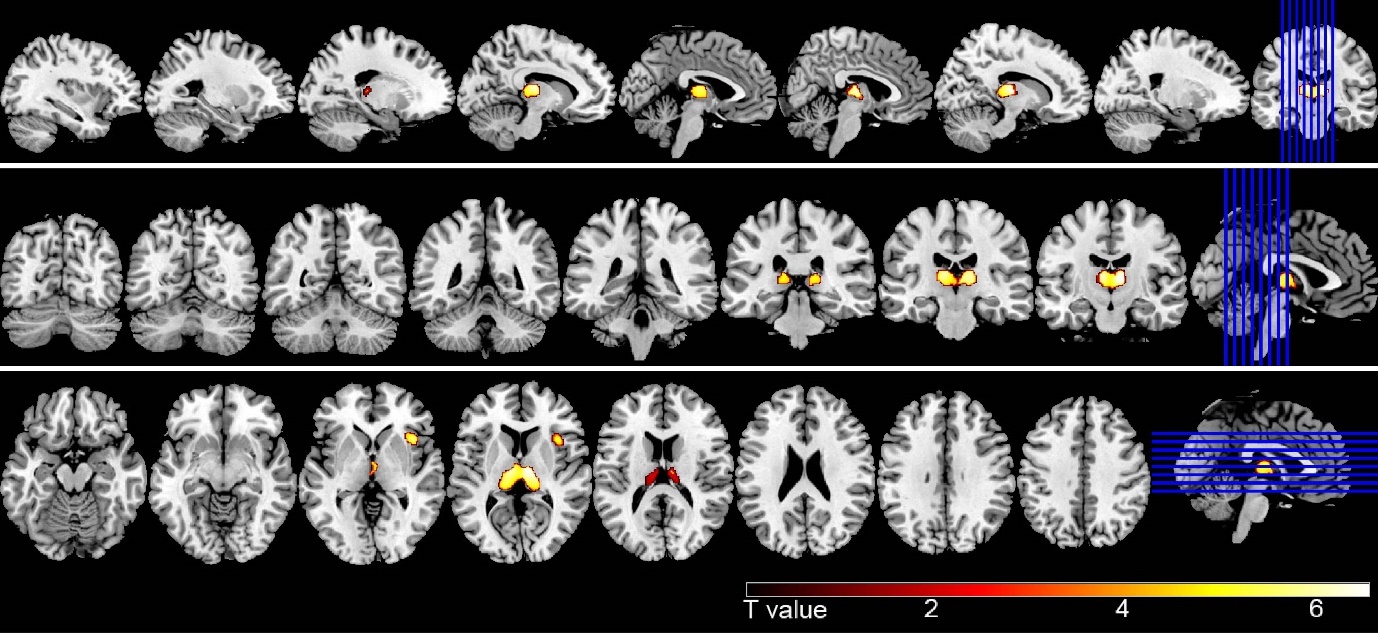


1. **Alpha**

**
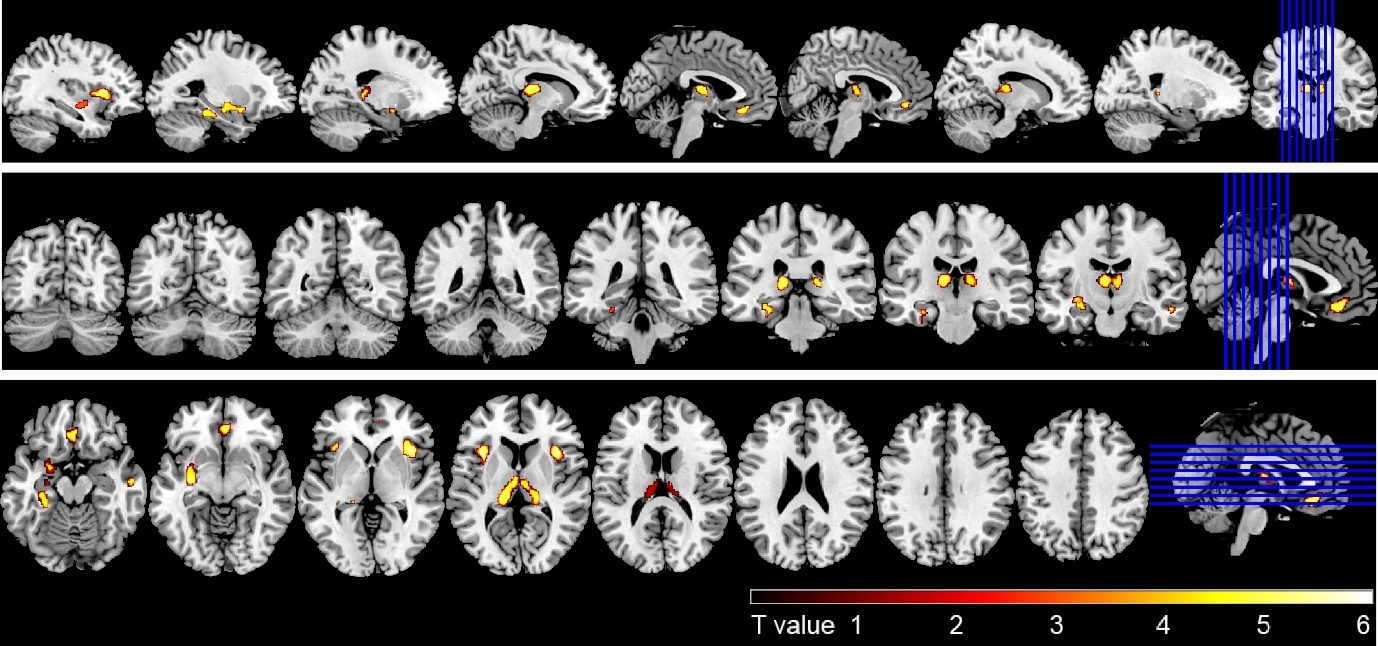
**

**Figure S2. Gray matter regions that were correlated with sedentary behavior parameters. Gray matter regions that were correlated with prolonged sedentary time (A), usual sedentary bout duration (B), and alpha (C).** The brain regions of reduced gray matter volume associated with prolonged sedentary time mainly involved the thalamus (A). The brain regions of reduced gray matter volume associated with usual sedentary bout duration mainly involved the right thalamus, right inferior frontal gyrus, and opercular part (B). The brain regions of reduced gray matter volume associated with alpha mainly involved the left thalamus, insula, left putamen, right middle temporal gyrus, right medial orbital part of the frontal lobe and left fusiform gyrus (C). The analyses were adjusted for age, sex, education, ActiGraph wear time and wear season, total intracranial volume, smoking, alcohol intake, and body mass index.

**References**

Chastin, S. F., & Granat, M. H. (2010). Methods for objective measure, quantification and analysis of sedentary behaviour and inactivity. *Gait Posture*, *31*(1), 82-86. <https://doi.org/10.1016/j.gaitpost.2009.09.002>

de Rezende, L. F., Rey-López, J. P., Matsudo, V. K., & do Carmo Luiz, O. (2014). Sedentary behavior and health outcomes among older adults: a systematic review. *BMC Public Health*, *14*, 333. <https://doi.org/10.1186/1471-2458-14-333>

Dempsey, P. C., Strain, T., Winkler, E. A. H., Westgate, K., Rennie, K. L., Wareham, N. J., Brage, S., & Wijndaele, K. (2022). Association of Accelerometer-Measured Sedentary Accumulation Patterns With Incident Cardiovascular Disease, Cancer, and All-Cause Mortality. *J Am Heart Assoc*, *11*(9), e023845. <https://doi.org/10.1161/jaha.121.023845>

Han, X., Jiang, Z., Li, Y., Wang, Y., Liang, Y., Dong, Y., Tang, S., Du, Y., & Qiu, C. (2021). Sex disparities in cardiovascular health metrics among rural-dwelling older adults in China: a population-based study. *BMC Geriatr*, *21*(1), 158. <https://doi.org/10.1186/s12877-021-02116-x>

Han, X., Song, L., Li, Y., Dong, Y., Liu, R., Han, Q., Wang, X., Mao, M., Cong, L., Tang, S., Hou, T., Zhang, Q., Liu, C., Han, X., Shi, L., Nyberg, L., Launer, L. J., Wang, Y., Du, Y., & Qiu, C. (2023). Accelerometer-Measured Sedentary Behavior Patterns, Brain Structure, and Cognitive Function in Dementia-Free Older Adults: A Population-Based Study. *J Alzheimers Dis*, *96*(2), 657-668. <https://doi.org/10.3233/jad-230575>

Han, X., Wang, X., Wang, C., Wang, P., Han, X., Zhao, M., Han, Q., Jiang, Z., Mao, M., Chen, S., Welmer, A. K., Launer, L. J., Wang, Y., Du, Y., & Qiu, C. (2022). Accelerometer-assessed sedentary behaviour among Chinese rural older adults: Patterns and associations with physical function. *J Sports Sci*, *40*(17), 1940-1949. <https://doi.org/10.1080/02640414.2022.2122321>

Liu, X., Li, Y., Li, L., Zhang, L., Ren, Y., Zhou, H., Cui, L., Mao, Z., Hu, D., & Wang, C. (2016). Prevalence, awareness, treatment, control of type 2 diabetes mellitus and risk factors in Chinese rural population: the RuralDiab study. *Sci Rep*, *6*, 31426. <https://doi.org/10.1038/srep31426>

Lu, J., Lu, Y., Wang, X., Li, X., Linderman, G. C., Wu, C., Cheng, X., Mu, L., Zhang, H., Liu, J., Su, M., Zhao, H., Spatz, E. S., Spertus, J. A., Masoudi, F. A., Krumholz, H. M., & Jiang, L. (2017). Prevalence, awareness, treatment, and control of hypertension in China: data from 1·7 million adults in a population-based screening study (China PEACE Million Persons Project). *Lancet*, *390*(10112), 2549-2558. <https://doi.org/10.1016/s0140-6736(17)32478-9>

Song, L., Han, X., Li, Y., Han, X., Zhao, M., Li, C., Wang, P., Wang, J., Dong, Y., Cong, L., Han, X., Hou, T., Liu, K., Wang, Y., Qiu, C., & Du, Y. (2023). Thalamic gray matter volume mediates the association between KIBRA polymorphism and olfactory function among older adults: a population-based study. *Cereb Cortex*, *33*(7), 3664-3673. <https://doi.org/10.1093/cercor/bhac299>

Song, P., Zha, M., Yang, X., Xu, Y., Wang, H., Fang, Z., Yang, X., Xia, W., & Zeng, C. (2019). Socioeconomic and geographic variations in the prevalence, awareness, treatment and control of dyslipidemia in middle-aged and older Chinese. *Atherosclerosis*, *282*, 57-66. <https://doi.org/10.1016/j.atherosclerosis.2019.01.005>

Wang, Y., Han, X., Zhang, X., Zhang, Z., Cong, L., Tang, S., Hou, T., Liu, C., Han, X., Zhang, Q., Feng, J., Yin, L., Song, L., Dong, Y., Liu, R., Li, Y., Ngandu, T., Kivipelto, M., Snyder, H.,…Qiu, C. (2022). Health status and risk profiles for brain aging of rural-dwelling older adults: Data from the interdisciplinary baseline assessments in MIND-China. *Alzheimers Dement (N Y)*, *8*(1), e12254. <https://doi.org/10.1002/trc2.12254>
